# Activation of cannabinoid receptor type 2 by JWH133 alleviates bleomycin-induced pulmonary fibrosis in mice

**DOI:** 10.18632/oncotarget.21975

**Published:** 2017-10-19

**Authors:** Qiang Fu, Yi Zheng, Xin Dong, Li Wang, Chun Guo Jiang

**Affiliations:** ^1^ Department of Rheumatology and Immunology, Beijing Chao-Yang Hospital, Capital Medical University, Beijing, China; ^2^ Department of Radiology, Beijing Chao-Yang Hospital, Capital Medical University, Beijing, China; ^3^ Department of Respiratory and Critical Care Medicine, Beijing Chao-Yang Hospital, Capital Medical University, Beijing, China

**Keywords:** cannabinoid receptor type 2, JWH133, pulmonary fibrosis, bleomycin, mice

## Abstract

Activation of cannabinoid receptor type 2 has been shown to have anti-fibrosis function in skin and heart. However, whether activating cannabinoid receptor type 2 inhibits pulmonary fibrosis remains elusive. Lung fibroblasts and TGF-β1 are key players in the pathogenesis of pulmonary fibrosis. In this research, we aimed to investigate the role of cannabinoid receptor type 2 in pulmonary fibrosis *in vitro* and *in vivo*. In lung fibroblasts stimulated by TGF-β1, preincubated by cannabinoid receptor type 2 agonist JWH133 not only reduced the elevated levels of collagen I and α-SMA, but also inhibited fibroblasts’ proliferation and migration. The dosage of JWH133 had no clear cytotoxic activity, and all these JWH133 effects were partially abrogated by cannabinoid receptor type 2 antagonist SR144528. In bleomycin-induced mice pulmonary fibrosis model, CT images of the lung tissue revealed an extensive ground-glass opacity, reticular pattern and fibrosis stranding. Notably, JWH133 treatment controlled the ongoing fibrotic process (showed by decreased lung density and fibrosis score). Meanwhile, lung histological results revealed that JWH133 treatment suppressed both the inflammatory response and extracellular collagen deposition. SR144528 may increase the pulmonary fibrosis, but no statistically significant difference was proved. Importantly, JWH133 reduced serum profibrotic cytokines levels of TGF-β1 and inhibited TGF-β1/Smad2 pathway *in vitro* and *in vivo*. Our research indicated that activating cannabinoid receptor type 2 by a pharmacological method might be a potential strategy for pulmonary fibrosis.

## INTRODUCTION

Pulmonary fibrosis is a group of diffuse parenchymal lung disorders. In addition to the idiopathic and specific environmental exposure, it is frequently associated with connective tissue disease [1]. Pulmonary fibrosis characterized by diffuse inflammation, interstitial fibrosis and irreversible destruction of lung architecture, which can be life-threatening [2]. Currently, no satisfied therapeutic method for pulmonary fibrosis has been established, in part because the disease mechanism is not fully understood [3]. Pulmonary fibrosis is initiated by micro-injury and inflammation followed by fibroblast activation. Those activated fibroblasts are more resistant to apoptosis and more likely differentiate to myofibroblast, which are considered to play a major role in fibrosis through abnormal transforming growth factor β1 (TGF-β1) production and excessive deposition of extracellular matrix [3–5].

Previous research revealed that endocannabinoids regulated a broad range of key cell signaling pathways: cell survival, invasion, metastasis and so on. Moreover, they exert anti-proliferative and anti-angiogenic effects in many different cancer models. Endocannabinoids exert their effects mainly through binding to two specific receptors: cannabinoid receptor type1 (CB1R) and cannabinoid receptor type 2 (CB2R) [6, 7]. The cannabinoid receptor type 1 is predominantly expressed in central nervous system and cannabinoid receptor type 2 (CB2R) has been initially detected in immune cells, but follow studies have identified CB2R in fibroblasts [8, 9]. Different studies prove that activating CB2R displays anti-inflammatory and anti-fibrogenic effects [7, 10–14]. In bleomycin induced skin fibrosis model, pharmacological manipulation of CB2R not only reduced dermal thickness and blood vessel’s collagen accumulation, but also prevented mast cell degranulation and macrophage infiltration in the skin, and all these effects abrogated by the CB2R antagonist AM630 [10]. CB2R-deficient mice were confirmed more sensitive to fibrosis progression [11]. Meanwhile, CB2R selective agonist AM1241, was reported to significantly improved cardiac function in myocardial interstitial fibrosis model and reduced the elevated levels of collagen I, collagen III and alpha-smooth muscle actin (α-SMA) [12].

However, research data regard for the role of cannabinoid receptor type 2 (CB2R) in lung fibrosis is limited. Considering that CB2R agonist has a strong potential as disease-modifying agents in fibrosis disease, we hypothesized activating CB2R might also be effective in preventing fibrosis in the experimental model of pulmonary fibrosis induced by bleomycin. To test this hypothesis, we investigated putative anti-fibrotic activity of cannabinoid receptor type 2 *in vitro* and *in vivo*.

## RESULTS

### Cannabinoid receptor type 2 agonist JWH133 inhibited TGF-β1 induced mice lung fibroblasts collagen I and α-SMA expression

**Figure 1:**
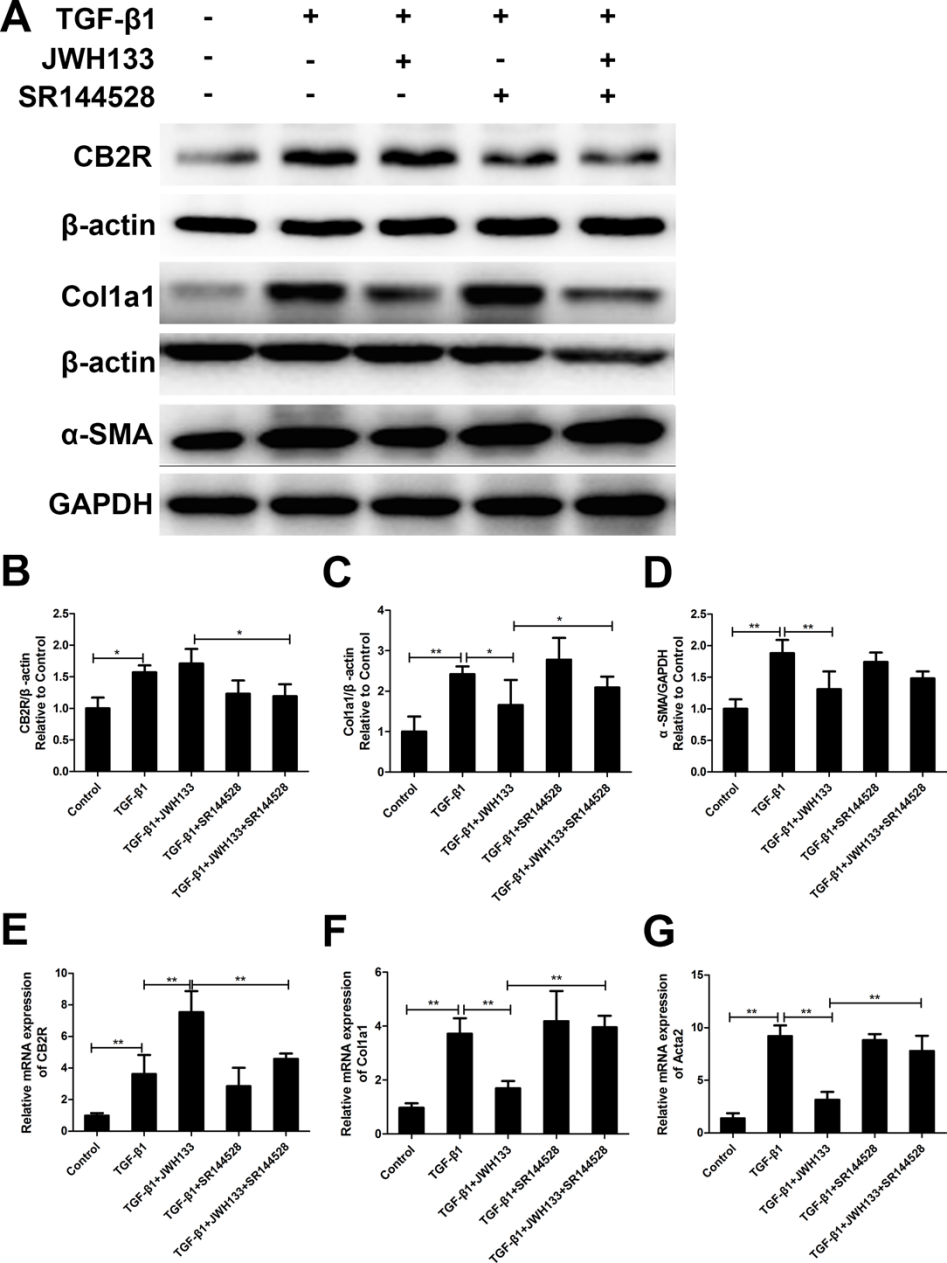
CB2R agonist JWH133 inhibited TGF-β1 induced mice lung fibroblasts collagen I and α-SMA expression Mice lung fibroblasts were preincubated (30 min, 37°C) with or without JWH133 (10μM) or/and SR144528 (1.0μM), then stimulated (48 h, 37°C) with TGF-β1 (5ng/ml). The expression of cannabinoid receptor type 2 (CB2R), collagen I (Col1a1) and α-SMA (Acta2) were evaluated by western blotting (**A**) and RT-qPCR (**E**, **F** and **G**); (**B**, **C** and **D)**: quantification of CB2R, Col1a1 and Acta2. Data are mean ± SD of 3 independent experiments. ^*^P < 0.05, ^**^P < 0.01.

We examined the effect of TGF-β1 stimulation on the expression of cannabinoid receptor type 2 (CB2R) in mice lung fibroblasts. The results revealed that TGF-β1 remarkably increased the protein and mRNA expression of CB2R in fibroblasts. Meanwhile, the RT-qPCR results revealed that CB2R mRNA levels were significantly increased in the TGF-β1+JWH133 group than in the TGF-β1 group (7.54 ± 1.33 vs. 3.62 ± 1.21, *P* < 0.01, Figure 1). As previous described, pulmonary fibroblasts activating and differentiate to myofibroblasts that can be triggered by TGF-β1 [4]. Therefore, as a specific marker for fibroblast activation and fibrosis, α-SMA and collagen I were examined. As shown in Figure 1, TGF-β1 meaningfully increased the protein expression of α-SMA and collagen I in cultured fibroblasts. Also, the RT-qPCR results revealed that α-SMA and collagen I mRNA levels were significantly increased in the TGF-β1 group than in the control group (9.21 ± 1.01 vs. 1.39 ± 0.48, and 3.71 ± 0.58 vs. 0.97 ± 0.17, both *P* < 0.01). Fibroblasts preincubated with cannabinoid receptor type 2 agonist JWH133 (30 min, 10 μM) resulted in lower mRNA and protein levels of α-SMA and collagen I (9.21 ± 1.01 vs. 3.14 ± 0.77, and 3.71 ± 0.58 vs. 1.69 ± 0.26, both *P* < 0.01, TGF-β1 group compared with TGF-β1+JWH133 group; Figure 1). Meanwhile, pre-treated with cannabinoid receptor type 2 antagonist SR144528 (30 min, 1.0 μM) reversed TGF-β1+JWH133 group trend in mRNA level, but not in protein level. These data suggest that JWH133 decreased TGF-β1 induced pulmonary fibrosis *in vitro*.

### Cannabinoid receptor type 2 agonist JWH133 inhibited TGF-β1 induced mice lung fibroblasts’ proliferation and migration.

**Figure 2:**
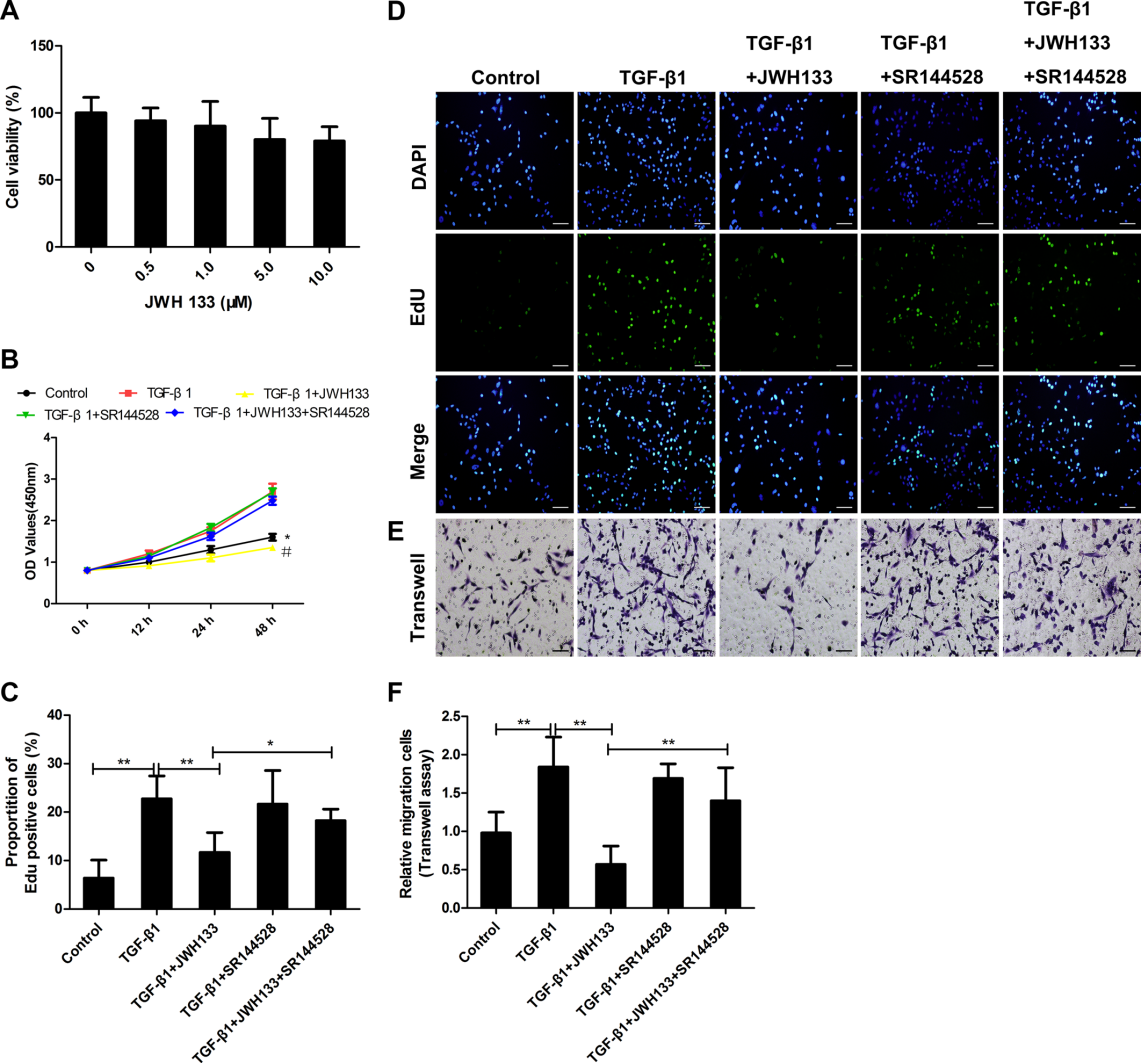
CB2R agonist JWH133 inhibited TGF-β1 induced mice lung fibroblasts proliferation and migration (**A**) JWH133 had no toxic effects on mice lung fibroblasts (MLF). MLF were treated with JWH133 at the indicated doses for 48 hours, and cell viability was analyzed by the MTT method. Results were expressed as percentage of cell viability against untreated cells. (**B**–**F**) MLF were preincubated (30 min, 37°C) with or without JWH133 (10μM) or/and SR144528 (1.0μM), then stimulated (24 h, 37°C) with TGF-β1 (5ng/ml); (B) Growth curve of MLF after intervention from hours 0 to 48. ^*^and^#^: *P* < 0.05 vs. TGF-β1 group; (C and D) The EdU assay showed that JWH133 could reduce TGF-β1-mediated MLF proliferation. (E and F) The MLF migration response to 10% FBS was analyzed using a Transwell assay. Cannabinoid receptor type 2 antagonist SR144528 could reverse TGF-β1+JWH133 group’s trend. Bar, 100 μm; Data are mean ± SD of 3 independent experiments. ^*^P < 0.05, ^**^P < 0.01.

We first want to investigate whether JWH133, the dosage we used in this study, have toxic effects on mice lung fibroblasts (Mlg2908). Mlg2908 cells were incubated with increasing concentrations of JWH133 for 24 h. Compared with untreated cells, the all range of JWH133 concentrations had little influence on cell viability (94 ± 9.50%, 90 ± 18.50%, 80 ± 15.61%, 79 ± 10.75%, both *P <* 0.05, Figure 2A), indicating that the research dosage of JWH133 had limited toxic effects on mice lung fibroblasts.

In addition, to establish the role of JWH133 in TGF-β1 induced mice lung fibroblasts growth, JWH133 (10 μM) was added to the culture medium beforehand. The CCK-8 method was used to assess the effects of JWH133 (10 μM) on the growth kinetics of Mlg2908. The growth curves shown in Figure 2B indicated that the growth ability of the Mlg2908 was decreased in the TGF-β1+JWH133 group compared with TGF-β1 group at 48h (1.35 ± 0.07 vs. 2.70 ± 0.19, *P <* 0.05). Furthermore, there were no differences between the TGF-β1 group and the TGF-β1+SR144528 group.

Meanwhile, the EdU proliferation assay (Figure 2C and 2D) suggested similar results. More EdU-positive cells were detected in the TGF-β1 group than in the control group (22.75 ± 4.70% vs. 6.38 ± 3.75%, *P* 0.01). And less EdU-positive cells were in the TGF-β1+JWH133 group compared with TGF-β1 group (11.67 ± 4.12% vs. 22.75 ± 4.70%, *P <* 0.01), suggesting that activation of CB2R inhibited TGF-β1 induced mice lung fibroblasts proliferation. However, preincubated with CB2R antagonist SR144528 reversed TGF-β1+JWH133 group trend (11.67 ± 4.12% vs. 18.23 ± 2.39%, *P <* 0.05).

We next investigated the effect of JWH133 on the migration capacity of mice lung fibroblasts induced by TGF-β1. As shown in Figure 2E and 2F, treating mice lung fibroblasts with TGF-β1 resulted in more cells to translocate through the insert chamber membrane. More relative migration cells were in the TGF-β1 group than in the control group (1.84 ± 0.39 vs. 0.98 ± 0.27, *P <* 0.01). Pretreatment with CB2R agonist JWH133 caused fewer cells found in the lower membrane, indicating that relative less migration cells were in the TGF-β1+JWH133 group compared with TGF-β1 group (0.57 ± 0.24 vs. 1.84 ± 0.39, *P <* 0.01) , suggesting that activation of CB2R inhibited TGF-β1 induced mice lung fibroblasts migration. Correspondingly, CB2R antagonist SR144528 reversed TGF-β1+JWH133 group trend (0.57 ± 0.24 vs. 1.40 ± 0.43, *P* < 0.01).

### Cannabinoid receptor type 2 agonist JWH133 prevents bleomycin-induced pulmonary fibrosis in mice.

As previously observed, C57BL/6 mice injected intratracheally for once with bleomycin can induce pulmonary fibrosis. In our research, we assessed the severity of mice lung fibrosis by using a new comprehensive evaluation method, including: computed tomography imaging, histological and biochemical methods. Especially, computed tomography images could provide information of the entire mice lung tissue.

**Figure 3:**
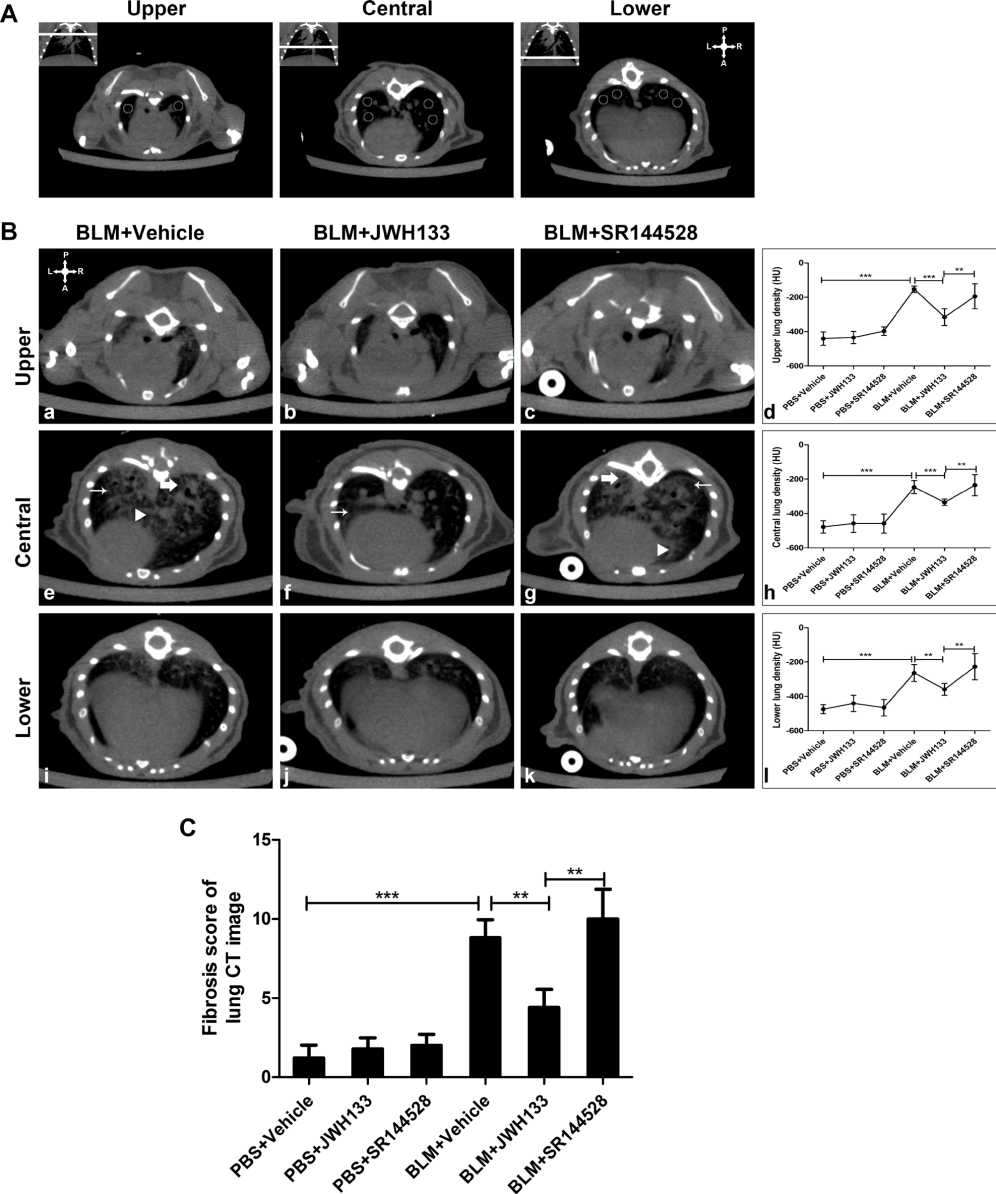
CB2R agonist JWH133 prevents BLM-induced computed tomography images of lung damage and fibrosis in mice (**A**) Lung density was expressed by Hounsfield units (HU) and 2 circles in the upper, 4 circles in the central and 4 circles in the lower region of the lung were measured. The upper-right symbol, gives the spatial position of posterior or dorsal (P), anterior or ventral (A), left (L) and right (R) regions. (**B**) Representative CT images of BLM administration mice at 21 days. Homographic slices, selected as described in figure (A), represent upper (a, b, c) central (e, f, g) and lower (i, j, k) pulmonary regions. Each slide of evaluation, including the following characteristic CT changes: ground-glass opacity (thin arrow), reticular pattern (bold arrow) and fibrosis stranding (triangle). Quantification of lung density in 6 group animals were assessed in upper (d) central (h) and lower (l) pulmonary regions. (**C**) Fibrosis score of mice lung CT image was also assessed. Image analysis was performed by two investigators independently and blindly. Graphs show mean ± SD of at least six mice for each group. ^*^P < 0.05, ^**^P < 0.01, ^***^P < 0.001.

The pulmonary densities were evaluated in representative slides from the upper, central and lower lung region (see in Figure 3A) at 21 days after PBS or bleomycin (BLM) administration. There were no significant differences between the PBS+Vehicle group, PBS+JWH133 group and PBS+SR144528 group in upper lung density (–440.48 ± 38.94, vs.–434.08 ± 35.72, vs.–397.00 ± 24.18, *P* < 0.05). In the central and lower lung region, the density evaluation displayed similar results (Figure 3d, 3h, and 3l). Compared to PBS+Vehicle group, BLM+Vehicle group mice showed an increased lung density in all upper, central and lower lung region (–440.48 ± 38.94, vs.–153.00 ± 18.08;–477.92 ± 35.46, vs.–246.71 ± 37.49;–474.33 ± 25.46, vs.–263.77 ± 48.74; both *P* < 0.001). To evaluate whether activating CB2R affects the development of bleomycin-induced pulmonary fibrosis, mice exposed to bleomycin were simultaneously treated with JWH133 (2.5 mg/kg). JWH133 reduced the pulmonary density in upper, central and lower lung region (BLM+JWH133 group vs. BLM+Vehicle group:–315.25 ± 49.48, vs.–153.00 ± 18.08;–334.56 ± 18.82, vs.–246.71 ± 37.49;–358.75 ± 34.31, vs.–263.77 ± 48.74; both *P* < 0.001). As showed in Figure 3 (a, e, and i), following 21 days of BLM instillation, CT images of the lung tissue revealed an extensive intralobular opacity, fibrosis stranding and honeycombing. Fibrosis score of mice lung CT image was also applied in Figure 3C. Preventive JWH133 co-treatment controlled the ongoing fibrotic process (BLM+JWH133 4.40 ± 1.14, vs. BLM+Vehicle 8.50 ± 1.06, *P* < 0.01). Meanwhile, BLM+SR144528 group mice showed higher lung density and fibrosis score to BLM+JWH133 group, but no significant differences to BLM+Vehicle group mice.

**Figure 4:**
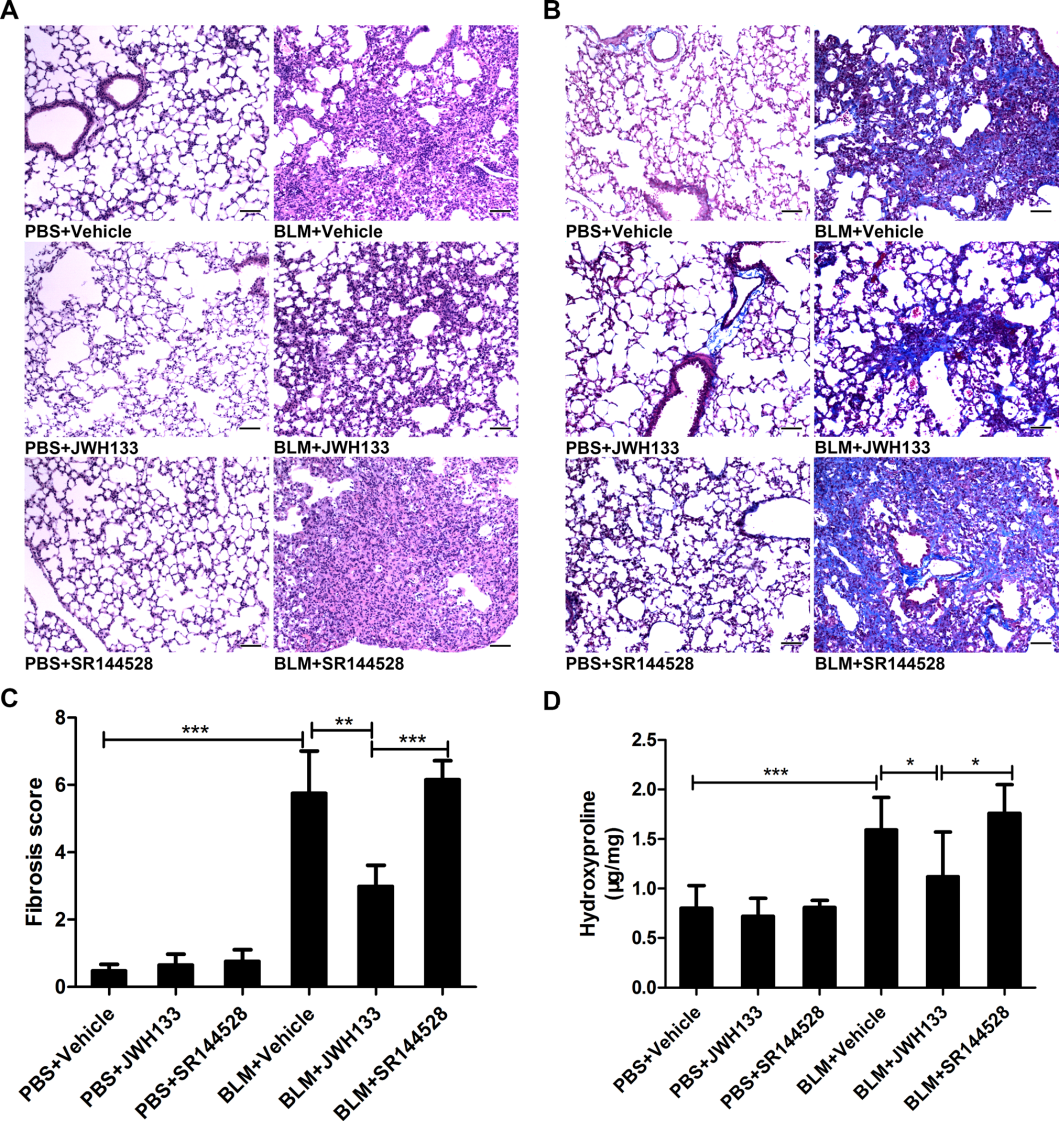
CB2R agonist JWH133 prevents bleomycin-induced lung damage and fibrosis in mice (**A** and **B**) Representative microphotographs (200 ×) of lung tissue slices stained by HE and Masson. (**C**) Semi-quantitative fibrosis scoring was assessed according to the Ashcroft scale. Slides were scored by a single investigator blindly. Bar, 100 μm; Graphs show mean ± SD of at least six mice for each group. (**D**) The hydroxyproline (Hyp) content of the pulmonary tissues was measured and presented as micrograms of Hyp per milligram of wet weight (μg/mg). ^*^P < 0.05, ^**^P < 0.01, ^***^P < 0.001.

Correspondingly, similar results were confirmed by histopathological examination of pulmonary biopsies stained with hematoxylin and eosin (HE) and Masson (Figure 4A and B). Preventive JWH133 co-treatment suppressed both the inflammation and fibrotic process, as showed by the Ashcroft score (BLM+JWH133 2.98 ± 0.63, vs. BLM+Vehicle 5.75 ± 1.25, *P* < 0.01, Figure 4C). As an important indicator of pulmonary fibrosis, the hydroxyproline content of the mice lung tissues has been measured (Figure 4D). Compared to BLM+Vehicle group, BLM+JWH133 group showed a decreased level of hydroxyproline in lung tissue (1.12 ± 0.45 μg/mg, vs. 1.59 ± 0.33 μg/mg; *P* < 0.05, Figure 4D). Those data indicated that JWH133 was sufficient to reduce the fibrotic process triggered by bleomycin.

**Figure 5:**
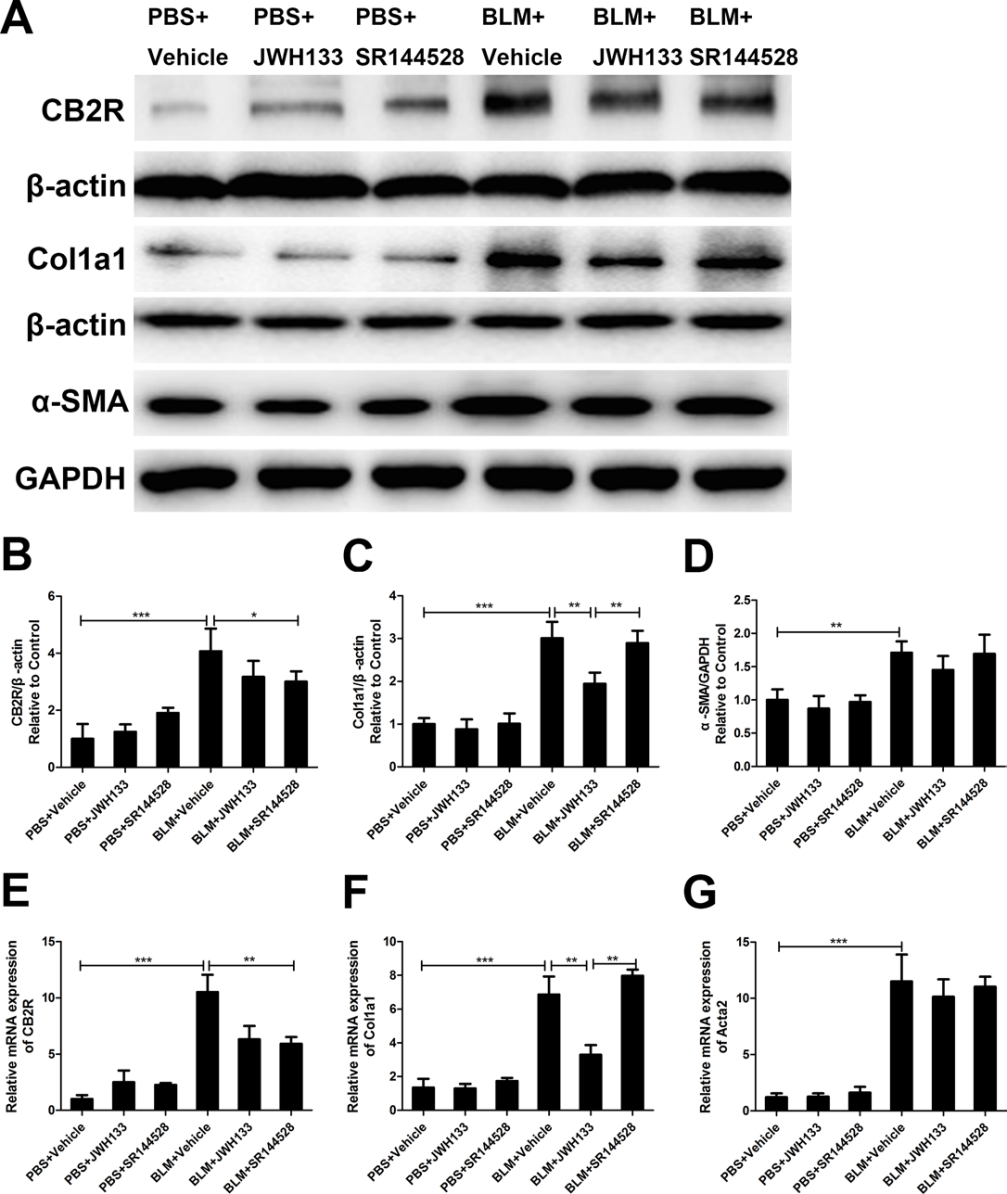
CB2R agonist JWH133 prevents bleomycin-induced collagen I expression in mice lung tissue The expression of cannabinoid receptor type 2 (CB2R), collagen I (Col1a1) and α-SMA (Acta2) were evaluated by western blotting (**A**) and RT-qPCR (**E**, **F** and **G**); **B**, **C** and **D**: quantification of CB2R, Col1a1 and Acta2. Graphs show mean ± SD of at least six mice for each group. ^*^P < 0.05, ^**^P < 0.01, ^***^P < 0.001.

The expression of cannabinoid receptor type 2 was prominently increased in the bleomycin-induced mice fibrosis model, and the protein and mRNA levels of CB2R in BLM+Vehicle group were significantly higher than those in PBS+Vehicle group (Figure 5). Furthermore, we tested the effect of JWH133 on the collagen I and α-SMA expression in fibrosis lung tissue. The results revealed that the protein and mRNA levels of collagen 1 and α-SMA were significantly increased following exposed to bleomycin compared with the PBS exposed group (Col1a1: 6.86 ± 1.08, vs. 1.35 ± 0.52; Acta2: 11.49 ± 2.40, vs. 1.21 ± 0.34; both *P* < 0.001, Figure 5). Moreover, preventive JWH133 co-treatment inhibited lung collagen I expression in mRNA (6.86 ± 1.08, vs. 3.30 ± 0.56, *P* < 0.01, Figure 5B) and protein levels. But, there were no significant differences between the BLM+Vehicle group and BLM+JWH133 group α-SMA expression in mRNA and protein levels.

### Cannabinoid receptor type 2 agonist JWH133 reduced serum profibrotic cytokines levels of TGF-β1 and inhibited TGF-β1/Smad2 pathway *in vitro* and *in vivo*.

**Figure 6:**
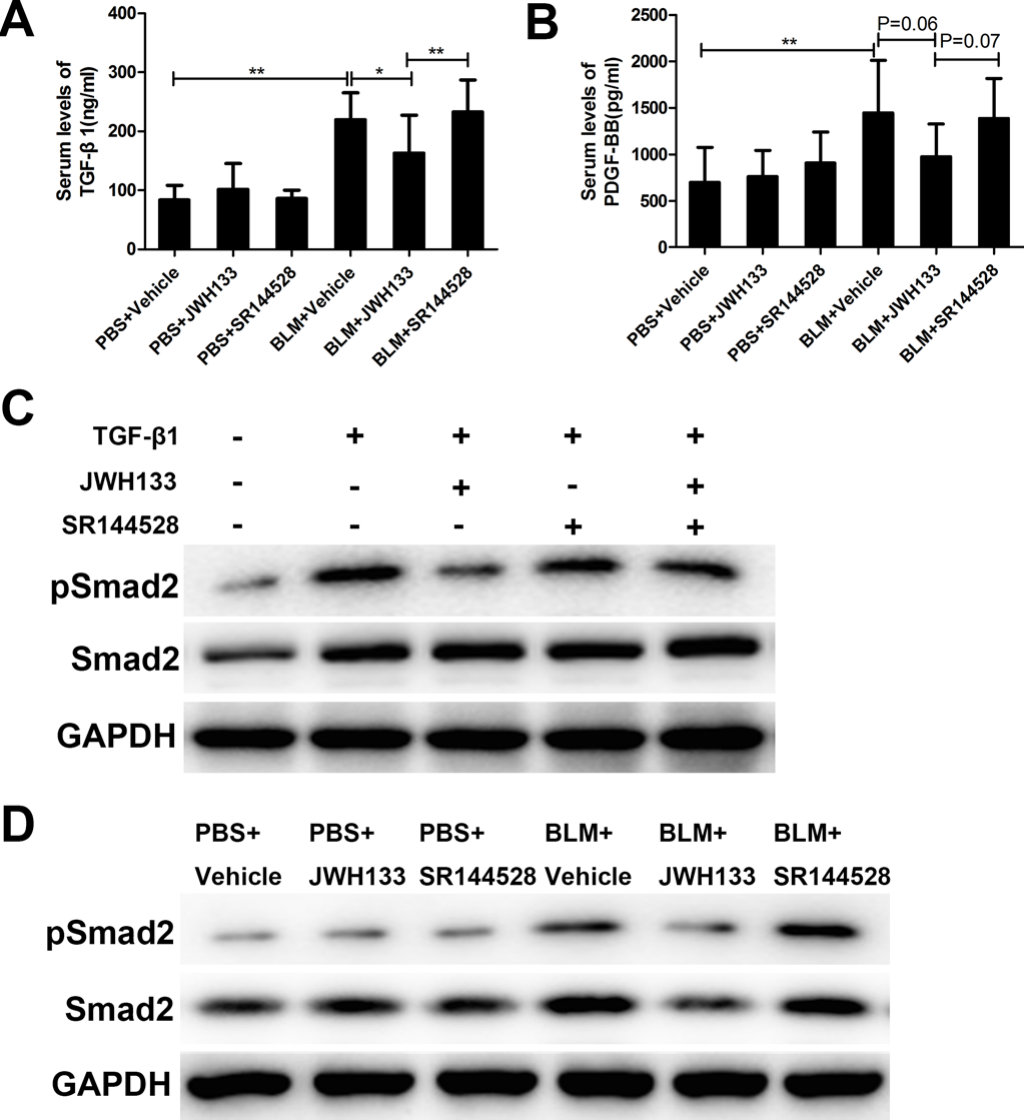
CB2R agonist JWH133 reduced the serum levels of profibrotic cytokines TGF-β1 in pulmonary fibrosis mice and decreased expression of Smad2 phosphorylation *in vitro* and *in vivo* (**A**) Serum levels of TGF-β1and (**B**) PDGF-BB were measured using ELISA method. Graphs show mean ± SD of at least six mice for each group. (**C**) MLF were preincubated (30 min, 37°C) with or without JWH133 (10μM) or/and SR144528 (1.0μM), then stimulated (1 h, 37°C) with TGF-β1 (5ng/ml). The results are representative of three independent experiments, as determined by western blot analysis. (**D**) The expressions of pSmad2 and Smad2 were evaluated by western blotting in lung tissue. ^*^P < 0.05, ^**^P < 0.01, ^***^P < 0.001.

Considering the important role of TGF-β1 and PDGF-BB in the pathogenesis of pulmonary fibrosis, we assessed their concentrations in the serum from mice. As previously observed, mice exposed to bleomycin significantly increased the serum levels of TGF-β1 and PDGF-BB compared with the PBS exposed mice (TGF-β1: 219.46 ± 64.31 ng/ml, vs. 83.45 ± 24.55 ng/ml; PDGF-BB: 1444.70 ± 564.46 pg/ml, vs. 696.30 ± 377.19 pg/ml; both *P* < 0.01, Figure 6). Additionally, the serum levels of TGF-β1 in the BLM+JWH133 group was significantly reduced compared with BLM+Vehicle group (219.46 ± 64.31 ng/ml, vs. 162.94 ± 41.60 ng/ml, *P* < 0.05). The level of PDGF-BB in the BLM+JWH133 group was also decreased compared with BLM+Vehicle group, but not statistical significantly (1444.70 ± 564.46 pg/ml, vs. 974.50 ± 351.85 pg/ml, *P* = 0.06, Figure 6B). Compared to BLM+JWH133 group mice, BLM+SR144528 group had higher serum level of TGF-β1 (232.81 ± 54.05 ng/ml, vs. 162.94 ± 41.60 ng/ml, *P* < 0.05), but no significant differences to BLM+Vehicle group. Excessive TGF-β/Smad signaling is the hallmark of fibrosis disease. To test the signaling pathways downstream of cannabinoid receptor type 2 activation, Smad2 phosphorylation was assessed *in vitro* and *vivo* by western blotting (see in Figure 6C and 6D). The results showed that JWH133 can reduce the Smad2 phosphorylation, which induced by TGF-β1 or bleomycin *in vitro* and *in vivo*.

## DISCUSSION

In the present study, we have shown that activation of cannabinoid receptor type 2 (CB2R) exerts anti-fibrotic effects *in vivo* and *in vitro*. We demonstrated that activating CB2R by selective agonist JWH133 is a potential strategy for the treatment of pulmonary fibrosis because it can inhibit TGF-β1 induced lung fibroblasts proliferation, migration, collagen I and α-SMA expression. In addition, JWH133 could efficiently prevent bleomycin-induced pulmonary fibrosis in mice.

The cannabinoid receptors control several central and peripheral functions, including neuronal transmission, cardiovascular functions, autoimmunity and inflammation [8]. They can also modulate cell motility and proliferation [8, 12, 15]. The expressions of CB1R and CB2R are increased in fibrosis disease, whereas CB1R and CB2R activations exert opposite effects: CB1R is profibrogenic and CB2R activation abrogates the fibrotic process [16, 17]. Consistent with our results, antifibrotic effects of activation CB2R has previously been observed in the skin, liver, kidney and myocardial infarction mice [9–12, 14, 16].

Fibroblasts are key players in the pathogenesis of fibrosis disease [4], and fibrosis is induced by activation, proliferation and migration of lung fibroblasts into the site of injury and followed by deposition of matrix proteins. Taking those into consideration, our study focused on lung fibroblast. We found that JWH133, a selective agonist of CB2R, reduced mice lung fibroblasts abnormal collagen I and α-SMA expression which induced by TGF-β1 *in vitro*. And the results in the bleomycin-induced pulmonary fibrosis mice model were similar *in vivo*. JWH133 treatment controlled the ongoing fibrotic process which proved by computed tomography imaging and histopathological methods. Meanwhile, JWH133 treatment inhibited collagen I expression in mRNA and protein levels. But, there were no significant differences in the α-SMA mRNA and protein levels between the BLM+Vehicle group and BLM+JWH133 group. Compared to pulmonary fibroblast *in vitro* experiments, lung tissue has a complex cellular composition. α-SMA is highly expressed in pulmonary vascular and bronchial smooth muscle cells, which may be the reason for no significant difference in α-SMA expression. In a recent work in myocardial interstitial fibrosis model, activation CB2R reduced the elevated levels of collagen I, collagen III and α-SMA [12]. VCE-004.8, an agonist of CB2R, inhibited TGF-β1 induced Col1A2 gene transcription and collagen deposition in skin fibrosis model [10].

Moreover, JWH133 significantly decreased the proliferation of pulmonary fibroblasts in our research. Correspondingly, in skin fibrosis experiment, not only the nonselective CB1R and CB2R agonist WIN-55,212 but also selective CB2 agonist JWH133 dramatically reduces the proliferation rate of skin fibroblasts *in vitro* [11]. CB2R agonists induced both growth arrest and apoptosis in myofibroblasts derived from cirrhotic liver [18]. Meanwhile, the previous research reported that selective activation of CB2R modulates chemotaxis of human monocytes [19]. In a murine model of dermal fibrosis, activation of CB2R inhibited the infiltration of leukocytes and macrophage into skin [10, 20]. Macrophage from CB2R-deficient mice had a significantly stronger migration potential towards the supernatant of apoptotic cardiomyocytes [21]. In line with this result, our research found that CB2R activation reduced lung inflammatory infiltration, which confirmed by histopathological analysis of pulmonary biopsies stained with hematoxylin and eosin (HE). Importantly, activation of CB2R not only limited inflammatory cell infiltration but also inhibited TGF-β1 induced mice lung fibroblasts migration. All these data indicate a direct role of the cannabinoid agonist on pulmonary fibroblasts to limit the fibrotic process.

TGF-β1 is a well-studied fibrotic cytokine and excessive TGF-β signaling is the hallmark of fibrosis disease. Previous studies have detected that CB2R activation attenuates the expression of TGF-β1 in myocardial fibrosis [15] and prevented myocardial fibrosis by promoting down-regulation of the TGF-β/Smad signaling pathway [12]. Phosphorylation of Smad2/3 was significantly down-regulated after synthetic cannabinoid WIN55, 212-2 exposure in skin fibrosis [22]. Consistent with previous results [12, 22], our research showed that activation of CB2R inhibited Smad2 phosphorylation *in vitro* and *vivo*. Therefore, TGF-β/Smad signaling pathway may be the downstream of CB2R activation. Meanwhile, we propose that activation of CB2R may inhibit resident lung fibroblast activation, proliferation and migration via inhibition of TGF-β1/Smad2 pathway. On the other hand, CB2R activation might exert its antifibrotic effects by regulating the secretion of fibrogenic cytokines. There are many important fibrogenic cytokines contributing to pulmonary fibrosis, including TGF-β1, platelet-derived growth factor (PDGF)-BB and so on. These molecules result in the activation and proliferation of lung fibroblasts [3–5]. Previous study demonstrated that CB2R activation by agonists ACEA and JWH133 selectively inhibited the release of VEGF and IL-6 from human lung macrophage [23]. The synthetic cannabinoid WIN55,212-2, a agonist of CB1R and CB2R, strongly inhibited TGF-β1, connective tissue growth factor (CTGF) and PDGF-BB expression in mice model of skin fibrosis induced by bleomycin [22]. In this study, we provided evidence that bleomycin induced a marked increase TGF-β1 and PDGF-BB levels in serum of mice. By contrast, JWH133 inhibited the expression of TGF-β1 in the serum of mice with pulmonary fibrosis. The level of PDGF-BB was also decreased, but not statistical significantly.

As far as we know, this is the first time to assess the antifibrotic effects of CB2R signaling by a comprehensive examination, including imaging, histological and biochemical methods in a bleomycin-induced pulmonary fibrosis mice model. In patients, noninvasive CT image follow-up is a general procedure to diagnose and monitor the development of lung fibrosis. Compared to conventional histopathological analysis, CT images could provide information in alive animals. Correspondingly, the image analysis results were paralleled to histopathological examination stained with HE and Masson in our study. Furthermore, CT images provided not only a direct angle for characteristic lung fibrosis changes, but also a chance for obtaining data at multiple time points from individual animals. However, the radiation dosage delivered to the mice during CT scan procedure might have influences on mice growth and progress in lung fibrosis [24]. Additional studies are necessary to investigate the animal micro-CT application in the pulmonary mice model.

We also investigated the effect of SR144528, a selective antagonist of CB2R, on the development of pulmonary fibrosis *in vitro* and *vivo*. Although SR144528 could reverse TGF-β1+JWH133 group’s trend in TGF-β1 induced mice lung fibroblasts proliferation and migration, SR144528 could not reverse the trend in α-SMA protein expression level *in vitro*. It’s probably because cannabinoids were pleiotropic and had multi-target activity in nature [8, 10]. Meanwhile, synergistic effect between TGF-β1 and SR144528 was also not observed *in vitro*. Importantly, imaging and histological results showed that SR144528 may increase the pulmonary fibrosis, but no statistically significant difference was proved *in vivo*.

Our study has several important limitations. The bleomycin-induced pulmonary fibrosis mice model is not irreversible. Another limitation is that this model mimics inflammation and fibrosis co-existence stage. Therefore, our findings indicate a potential prevented role of activating CB2R in the early stage of pulmonary fibrosis disease. Additional studies are necessary to investigate the role of CB2R in the later fibrosis stage and its’ therapeutic effect.

In conclusion, we demonstrate that activating cannabinoid receptor type 2 by selective agonist JWH133 is a potential strategy for pulmonary fibrosis. Our researches offer a new choice for this life-threatening disease.

## MATERIALS AND METHODS

### Cell cultures

Mice lung fibroblasts (Mlg2908) were purchased from American Type Culture Collection (ATCC, Manassas, USA). Cells were cultured in DMEM medium (Gibco) , and supplemented with 10% fetal bovine serum (FBS, Gibco), 1% penicillin/streptomycin at 37°c in a humidified atmosphere containing 5% CO_2_. The cells were treated with different combinations of vehicle (DMSO), JWH133 (cannabinoid receptor type 2 agonist; Tocris Bioscience, Bristol, UK), SR144528 (cannabinoid receptor type 2 antagonist; Tocris Bioscience, Bristol, UK) and transforming growth factor β1 (TGF-β1; R&D Systems Inc. Minneapolis, USA).

### Cytotoxicity assays

For the MTT (Sigma, USA) test, Mlg2908 cells were seeded at a density of 1000 cells/well in 96-well plates and incubated with increasing concentrations of JWH133 for 48 hours as previously described [25]. The absorbance was measured in a microtiter plate reader (Thermo Fisher Scientific, Waltham, MA, USA) set to 570nm, and the untreated controls were considered as 100% survival.

### Cell proliferation assay

The cells were treated with different combinations of vehicle, TGF-β1 (5 ng/ml), JWH133 (10 μM) and SR144528 (1.0 μM). For the cell counting kit-8 (CCK-8, Beyotime Institute of Biotechnology, China) test, 5000 cells/well were plated onto 96-well plates in a triplicate pattern. Assays were performed from 0 to 48 hours after plating by the addition of 100μl of fresh medium in 10μl of the CCK-8 solution for another 4 h at 37 °C as previously described [25]. The absorbance was measured in a microtiter plate reader (Thermo Fisher Scientific, Waltham, MA, USA) set to 570nm, and the assay was repeated three times.

The 5-ethynyl-2’-deoxyuridine (EdU) assay (RIBOBio Co, Guangzhou, China) was used to measure cells’ ability to proliferate after treatment with different combinations of vehicle, TGF-β1 (5 ng/ml), JWH133 (10 μM) and SR144528 (1.0 μM) for 24 h. After incubation with EdU for 2 h, the cells were fixed with 4% paraformaldehyde and permeabilized with 0.5% Triton X-100. Then, the Apollo^®^ reaction cocktail (reaction buffer and Apollo^®^ 567 fluorescence) was added to the medium for another 30 min in the dark. After being washed with PBS 3 times, the cells were stained with DAPI (Sigma-Aldrich) for 5 min and immediately viewed under fluorescence microscopy. The number of EdU positive cell was calculated by counting at least three random separate fields.

### Migration assays

The migration assay was performed using a 24-well transwell chamber with a pore size of 8 μm (Corning, USA). Mlg2908 were cultured with different combinations of vehicle, TGF-β1 (5 ng/ml), JWH133 (10 μM) and SR144528 (1.0 μM) for 24 h. Then, 1 × 10^4^ cells were seeded into the upper chamber in serum-free medium. 10% fetal bovine serum (FBS) was added to the lower chamber. After 10h incubation at 37°C, the non-migrating cells in the upper chamber were carefully removed with a cotton swab, and the cells that had traversed the membrane were fixed in methanol and stained with 0.05% crystal violet as previously described [25]. The number of migrated cells was calculated by counting at least three random separate fields.

### Reverse transcription quantitative real-time polymerase chain reaction (RT-qPCR)

Total RNA was extracted from Mlg2908 cells and mice pulmonary tissue samples using the simple Total RNA kit (Tiangen Biotech Co., Ltd., Beijing, China) according to the manufacturer’s instructions. RNA was reverse transcribed using the Revert Aid First Strand cDNA Synthesis kit (Takara Biotechnology Co., Ltd., Dalian, China) and quantitative PCR was performed using the SYBR PrimeScript RT-PCR kit (Takara Biotechnology Co., Ltd., Dalian, China).

The process of RT-qPCR was implemented by ABI Prism 7500 (Applied Bio-systems, Foster City, CA, USA) with the pre-designed primers for *Col1a1* (forward: 5′-TGACTGGAAGAGCGGAGAGT-3′; reverse: 5′-GACGGCTGAGTAGGGAACAC-3′), *Acta2* (forward:5′-GTCCCAGACATCAGGGAGTAA-3′; reverse: 5′-TCGGATACTTCAGCGTCAGGA-3′), *CB2R* (forward: 5′-TGGACCTGGGTGACTGG-3′; reverse: 5′-CATCTGGGATACCTGAAACA-3′) and *Gapdh* (forward: 5′-AAGACCCAGAAATGAAC-3′; reverse: 5′-TCTACACGATAACAACCA-3′).

The level of gene expression was quantified using the formula 2^-ΔΔCT^ to calculate the relative expression levels of genes. Both samples were examined in triplicate, and the experiment was repeated three times.

### Western blot analysis

Cells or lung tissues were collected, and total protein was extracted. The following antibodies were used: β-actin (1:500; sc-130301; Santa Cruz Biotechnology, Inc., CA, USA), GAPDH (1:1000; sc-47724; Santa Cruz Biotechnology, Inc., CA, USA), collagen 1 (1:2000; ab34710, Abcam, Cambridge, UK), α-SMA (1:500; ab5694, Abcam, Cambridge, UK), CB2R (1:500; ab3561, Abcam, Cambridge, UK), pSMAD2 (1:1000; 3104, Cell Signaling Technology, Inc., MA, USA), SMAD2 (1:1000; 5339, Cell Signaling Technology, Inc., MA, USA) and the fluorescent-labeled secondary antibodies (1:10,000; 600-101-096; Rockland, Inc., Gilbertsville, PE, USA). The bands were visualized using a double-infrared laser scanning imaging system (LI-COR Biosciences, Lincoln, NE, USA).

### Animal experiments

Seven-week-old male C57BL/6 mice (body weight, 20-22 g; Vital River Laboratories, Beijing, China) were used in all experiments. The animals were maintained under specific pathogen-free conditions (temperature 22˚C ± 3˚C; 12 h light/dark cycle and relative humidity of 50% ± 10%) with free access to standard food and water. All procedures involving animals were approved by the Animal Experiments and Experimental Animal Welfare Committee of Capital Medical University (Beijing, China). For the model of pulmonary fibrosis as previously described [26, 27], mice underwent intratracheal bleomycin instillation (5.0 mg/kg bleomycin (Nippon Kayaku Co., Ltd., Tokyo, Japan) in 100μl phosphate-buffered saline); the control animals were injected intratracheally with the same volume of phosphate-buffered saline (PBS). On day 1 following bleomycin instillation, mice were treated in parallel by daily intraperitoneal injection of CB2 agonist JWH133 (2.5 mg/kg), CB2 antagonist SR144528 (1 mg/kg) or vehicle. JWH133 and SR144528 were purchased from Tocris (Bristol, UK). The mice were randomly divided into 6 groups as follows: PBS + Vehicle, PBS + JWH133, PBS + SR144528, BLM + Vehicle, BLM + JWH133, BLM + SR144528. Six to eight animals were analyzed per experimental group. At the designated time points (21 days after bleomycin administration), the mice were fulfilled lung imaging examination, and then humanely sacrificed by an overdose of the anesthesia (inhalation of isoflurane) for serum and histological measurements.

### Computed tomography scanning

All computed tomography (CT) images were acquired using Siemens Inveon Micro-CT scanner (Erlangen, Germany); 80 kVp with 500μA were applied. Thin-section slices, each 0.078 mm in thickness, and field of view 30 × 30 mm, matrix 512 × 512, acquisition time one second were performed. Around 140 to 150 slices covered the entire mice lungs. Mice were anesthetized by inhalation of isoflurane during the scan. Images were visualized using Materialise's interactive medical image control system 10.01 (Belgium). Image analysis was performed by two investigators independently and blindly.

The pulmonary densities were evaluated as described previously [24, 28], with some modifications. Representative slides from the upper, central and lower lung region were measured quantitatively by figure out Hounsfield units (HU). Window and level setting for all images were the same. Average HU of the upper, central and lower lung region was calculated separately. Main bronchi and vessels were omitted. Ten circles of interest were selected in the following areas of the left and right lungs: upper region (Figure 3A; about 25 slides below the main bronchi enter the lung), central region (about 80 slides below the main bronchi enter the lung) and lower lung region (about 120 slides below the main bronchi enter the lung). These circles were of two mm^2^.

Fibrosis score of mice lung CT image was also applied with some modifications [28, 29]. A 5-point ordinal scale for radiographs: grad 0, indicating no changes of tissue, no change of opacity and fibrosis; grade 1, indicating mild changes, involving less than 25% of the lung; grade 2, indicating moderate changes, involving 25% to 50% of the lung; grade 3, indicating severe changes, involving less than 75% of the lung; grade 4, indicating maximal changes, involving more than 75% of the lung. Each slide of evaluation, including the following characteristic CT changes: ground-glass opacity, reticular pattern and fibrosis stranding. Representative slides from the upper, central and lower lung region were measured separately, and a total lung fibrosis score was calculated as the sum of the three items scores.

### Hydroxyproline assay

The hydroxyproline contents in lungs of each group were measured with a conventional hydroxyproline method [30]. The ability of the assay to completely hydrolyze and recover hydroxyproline from collagen was confirmed using samples containing known amounts of purified collagen. Absorbance was measured at a wavelength of 550 nm and the amount of hydroxyproline was determined.

### Histological examination

Lung samples were fixed in 4% paraformaldehyde (Solarbio, Beijing, China) for histological examination. After 24 h, the tissues were dehydrated and embedded in paraffin. Paraffin Sections 2–4 μM thick were cut from fixed lungs, stained with hematoxylin and eosin (HE) and Masson’s reagent (Solarbio, Beijing, China), and examined with a microscope. The histological severity of pulmonary fibrosis was scored as described in the study by Ashcroft et al. [31].

### Enzyme-linked immunosorbent assay

Serum levels of transforming growth factor β1 (TGF-β1) and platelet-derived growth factor BB (PDGF-BB) were measured using specific ELISA kits from R&D Systems Inc. (Minneapolis, Minnesota, USA) according to the manufacturer’s instructions.

### Statistical analysis

The results were expressed as mean ± SD and analyzed using a statistical software package (SPSS 17.0; SPSS, Inc., Chicago, IL, USA). Statistical analysis was performed using one-way analysis of variance (ANOVA) followed by a Scheffe post-hoc test. Values at P<0.05 were considered statistically significant.
